# A longitudinal study on α-synuclein in blood plasma as a biomarker for Parkinson's disease

**DOI:** 10.1038/srep02540

**Published:** 2013-08-29

**Authors:** Penelope G. Foulds, Peter Diggle, J. Douglas Mitchell, Angela Parker, Masato Hasegawa, Masami Masuda-Suzukake, David M. A. Mann, David Allsop

**Affiliations:** 1 Division of Biomedical and Life Sciences, Faculty of Health and Medicine, University of Lancaster, Lancaster, LA1 4AY, UK; 2 Lancaster Medical School, Faculty of Health and Medicine, University of Lancaster, Lancaster, LA1 4AY, UK; 3 Royal Preston Hospital, Sharoe Green Lane, Preston PR2 9HT, UK; 4 Department of Neuropathology and Cell Biology, Tokyo Metropolitan Institute of Medical Science, 2-1-6 Kamikitazawa, Setagaya-ku, Tokyo, 156-8506, Japan; 5 Centre for Clinical and Cognitive Neuroscience, Institute of Brain, Behaviour and Mental Health, University of Manchester, Salford Royal Hospital, Salford, M6 8HD

## Abstract

There have been no longitudinal studies on α-synuclein as a potential biomarker for the progression of Parkinson's disease (PD). Here, blood plasma ‘total α-synuclein’ and ‘Ser-129 phosphorylated α-synuclein’ were assayed at 4–6 monthly intervals from a cohort of 189 newly-diagnosed patients with PD. For log-transformed data, plasma total α-synuclein levels increased with time for up to 20 yrs after the appearance of initial symptoms (p = 0.012), whereas phosphorylated α-synuclein remained constant over this same period. The mean level of phosphorylated α-synuclein, but not of total α-synuclein, was higher in the PD plasma samples taken at first visit than in single samples taken from a group of 91 healthy controls (p = 0.012). Overall, we conclude that the plasma level of phosphorylated α-synuclein has potential value as a diagnostic tool, whereas the level of total α-synuclein could act as a surrogate marker for the progression of PD.

Parkinson's disease (PD) is a neurological movement disorder that has been estimated to affect more than 6 million people worldwide. The defining neuropathological features of idiopathic PD are the loss of dopaminergic neurons from the substantia nigra (SN) and the presence of Lewy bodies (LBs) and Lewy neurites (LNs) in surviving neurons of this and other mid brain (and brain stem) regions^1^. The drugs currently available for PD can partially compensate for this loss of dopaminergic neurones, but there is no treatment that can prevent or slow the progression of this disease.

LBs and LNs both contain fibrils composed of α-synuclein (α-syn)^1^,^2^. Pathological changes involving α-syn are also found in two other ‘α-synucleinopathies’, namely dementia with Lewy bodies (DLB) and multiple system atrophy (MSA)^3^. Duplication, triplication and mutation of the *SNCA* gene are all causes of hereditary forms of either PD or DLB^4^. Wild-type or mutant α-syn, when overexpressed in transgenic animals, produces a phenotype resembling PD^5^,^6^ and soluble oligomers derived from α-syn are toxic to nerve cells^7^. These observations suggest that α-syn plays a pivotal role in the development of the α-synucleinopathies.

A correct diagnosis of PD is critically dependent on clinical examination to rule out other movement disorders, such as essential tremor and progressive supranuclear palsy (PSP), which may initially mimic this disease^8^. Although the positive predictive value of the final clinical diagnosis of idiopathic PD when made by a movement disorder specialist can be very high (up to 98.6% in some cases)^9^, diagnosis in the early stages is far less certain, with almost one third of the clinical diagnoses in patients with parkinsonism being revised within, on average, the first five years of disease onset^9^. Hence, a readily accessible molecular diagnostic biomarker for PD, particularly one that is applicable at the early stage of the illness, would be of major benefit. A molecular biomarker that could track disease progression in already-diagnosed patients with PD would be useful in clinical trials of new disease-modifying drugs.

α-Syn is present in human body fluids including blood plasma^10^ and has attracted considerable interest in recent years as a potential molecular biomarker for PD. This extracellular form of α-syn could play an important role in a prion-like cell-to-cell transfer of α-syn pathology in the brain^11^,^12^. There is an emerging consensus that the level of ‘total α-syn’ is, on average, lower in samples of CSF taken from a group of patients with PD than in groups of normal or neurological controls^13^,^14^,^15^. However, obtaining CSF is an invasive procedure, and analysis of α-syn levels in CSF is not amenable to longitudinal study. There have been some studies of α-syn as a potential biomarker in the much more accessible peripheral blood, with an initial report suggesting increased levels of this protein in plasma samples from patients with PD compared with those from healthy controls^16^. However, subsequent studies have reported decreased levels of α-syn in plasma from individuals with PD^17^ or no significant change^18^.

We have set up a longitudinal study in almost 200 newly diagnosed patients with PD to examine how the levels of different forms of α-syn in blood plasma might change with disease progression. In an earlier report of results from the initial phase of this study^19^, we developed and employed immunoassays for total and oligomeric forms of both normal and phosphorylated (at Ser-129) α-syn to measure the levels of these different forms of the protein in plasma samples from the first 32 patients enrolled onto our longitudinal study. This initial report was based on blood samples taken at months 0, 1, 2 and 3 from these 32 PD cases, as well as single plasma samples from a group of 30 healthy control participants. We found that the levels of α-syn in plasma varied greatly between individuals, but were remarkably consistent over time in repeat samples obtained from each individual with PD. The mean level of phosphorylated α-syn was found to be numerically higher in the PD samples than in the controls, but this difference did not reach statistical significance (p = 0.053). Thus, we reported that while Ser-129 phosphorylated α-syn shows some initial promise as a diagnostic marker for PD, this needs to be confirmed in larger scale studies^19^. Because α-syn accumulates in a phosphorylated form in LBs^2^, it is possible that this modified, pathological form of the protein will more accurately reflect the fundamental neuropathology of PD than previous measures of ‘total α-syn’^19^,^20^. Here, we report the results obtained from the second phase of our longitudinal study, undertaken over a more extended time period of up to 4 years from initial recruitment, to consolidate to what extent α-syn can act as a diagnostic marker, and to determine whether it can act as a marker of disease progression.

## Results

### Patient population and demographics

Demographic details of the cohort of 189 patients with PD that was followed at 4–6 months intervals following initial recruitment are summarized in table 1. The mean age of this cohort on ascertainment and initial sampling was 61.9 yr (youngest 39 yr, oldest 82 yr) and there were 119 males and 70 females. Among the 91 recruited healthy controls, there were 32 males and 59 females, with a mean age of 65.3 yr (youngest 42 yr, oldest 82 yr). The PD cases and control subjects were recruited in parallel, at the same clinical centres, and the blood samples were taken and processed in the same way and by the same personnel at each participating site. Moreover, the plasma samples were randomized for analysis, with both control and PD samples being assayed together on the same microtitre plates.

### Immunoassay results

**Figure 1:**
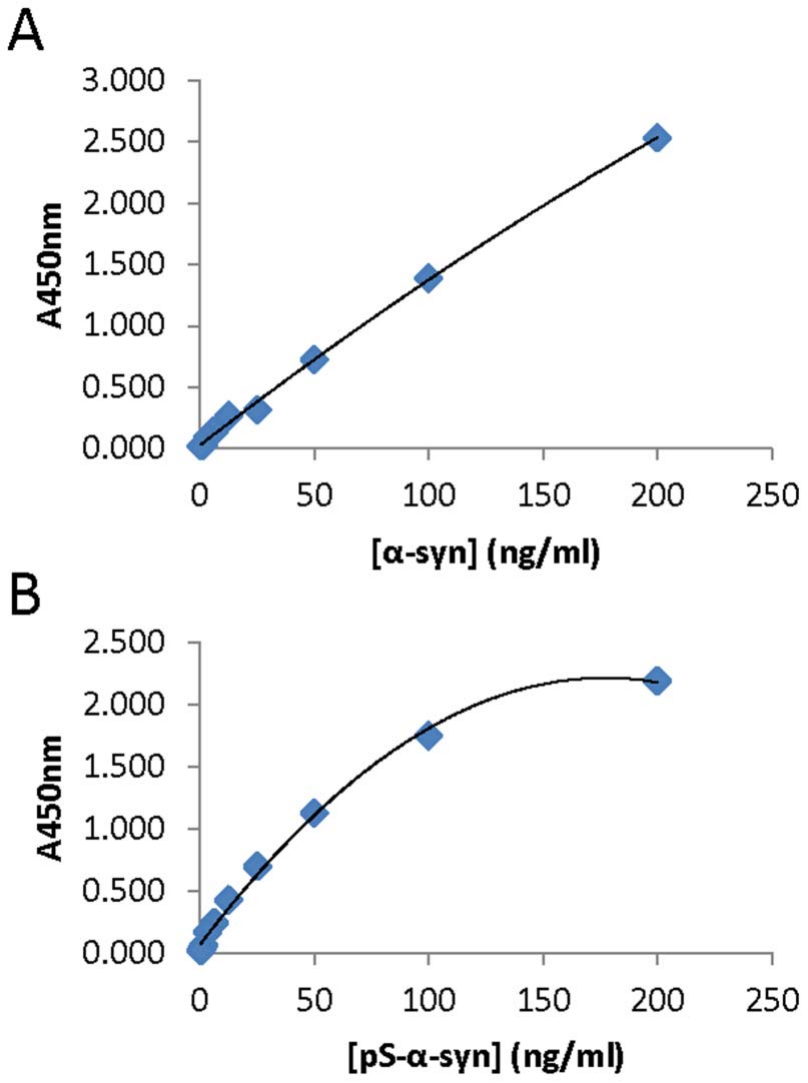
Examples of standard curves obtained for total α-syn (A) and phosphorylated α-syn (B). These are representative curves, each obtained from a single ELISA plate.

**Figure 2:**
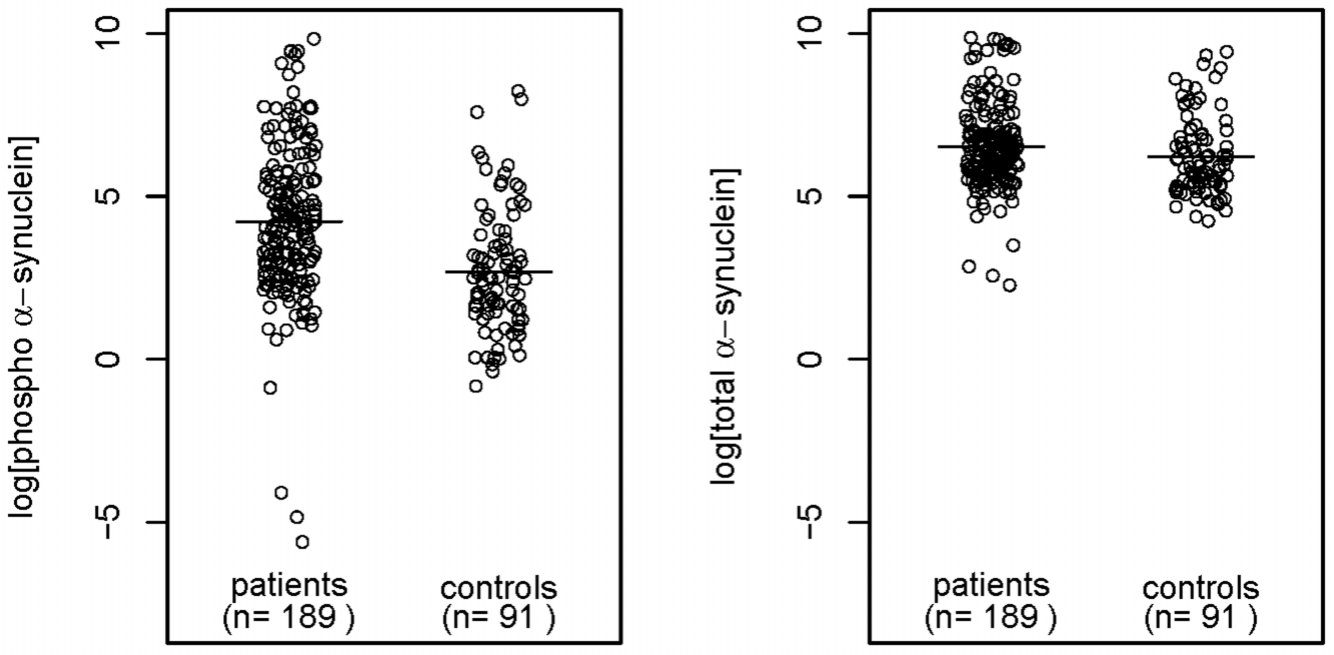
Baseline measurements of log-transformed α-syn plasma concentrations in PD patients and normal healthy controls. Left-hand panel shows log-transformed phosphorylated α-syn, right-hand panel log-transformed total α-syn, horizontal lines denote sample means.

Figure 1 shows representative examples of standard curves for each of the two different immunoassays. Empirical distributions of the plasma α-syn concentrations for both assays were highly skewed on the original scale, and so log-transformed data were used for all of the cross-sectional and longitudinal statistical analyses described below. Figure 2 presents dot-plots of the baseline data on PD cases and controls pertaining to each assay. In the case of cross-sectional analysis of the PD samples, we have used the blood sample taken at the first visit as the baseline value, whereas data for single blood samples only were available from the controls. To investigate the potential of α-syn as a means of discriminating between patients with PD and healthy controls, we determined whether there was any significant difference between the average level of α-syn (on the logarithmic scale) across patients and controls, within the two different α-syn assays. Using a standard two-sample *t* test on log-transformed concentrations, there was found to be no significant difference between the average levels of PD patients and healthy controls for ‘total α-syn’ (p = 0.058), whereas the average level of phosphorylated α-syn was found to be significantly higher for the PD patients than for the healthy controls (p = 0.012). The mean levels for each assay, on the original scale, are given in table 1. It can be seen that the mean level of phosphorylated α-syn was approximately 5-fold higher in the PD samples (756.8 ng/ml) than in the healthy controls (143.4 ng/ml).

**Figure 3:**
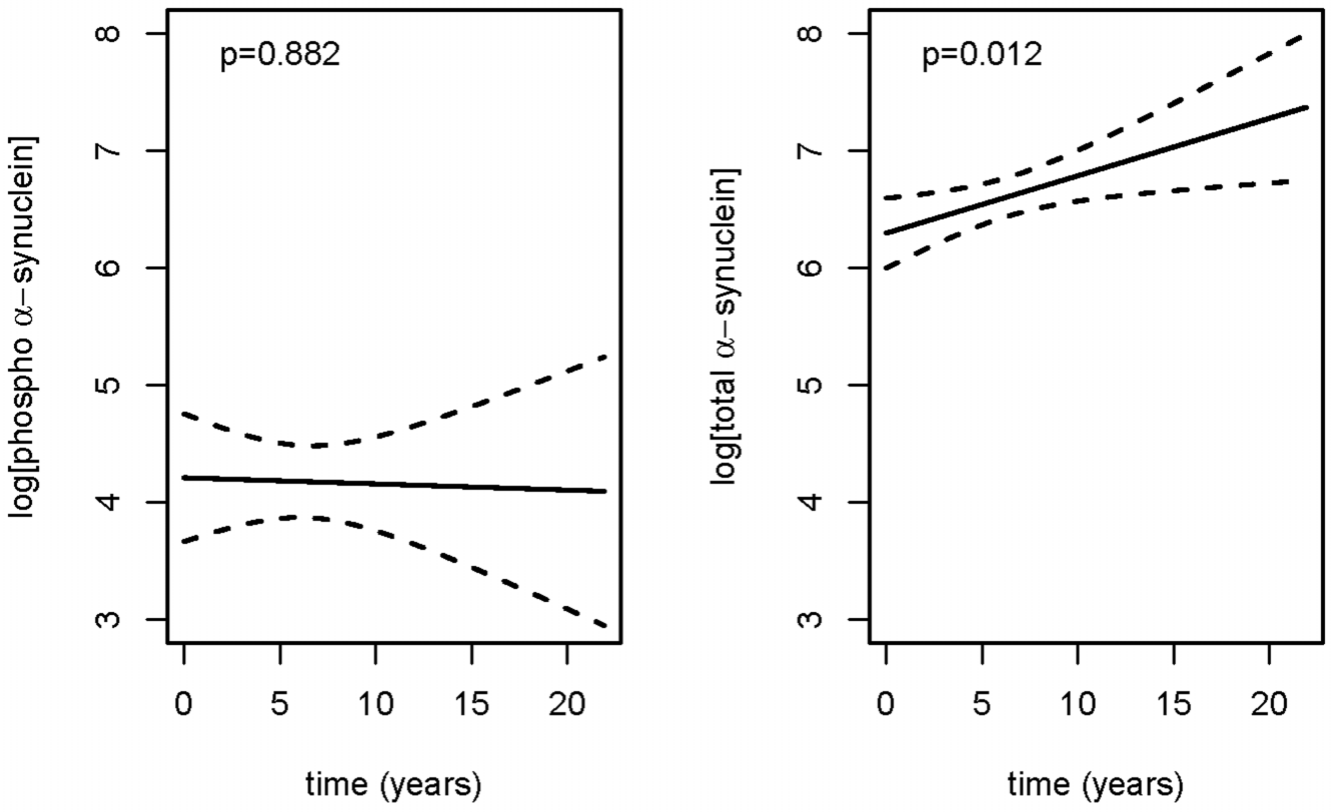
Fitted time-trends (solid lines) and 95% pointwise confidence intervals (dashed lines) for repeated measurements of log-transformed α-syn plasma concentrations obtained from patients with PD, plotted as time since onset of initial symptoms. P-values refer to Wald tests of the hypothesis of no time-trend. Note that the level of phosphorylated α-syn does not change significantly with disease progression, whereas total α-syn shows a significant increasing time-trend.

The longitudinal data for the immunoassay results from the PD patients are shown in figure 3 which presents the estimated time-trends together with 95% pointwise confidence intervals for the two different measures of α-syn, onwards from the time (t = 0) of reported appearance of initial symptoms (not necessarily, therefore, from time of initial diagnosis). We have chosen to plot the results in this way so that any changes in the levels of total α-syn and phosphorylated α-syn as the disease progresses over time can be best appreciated. Some patients were recruited onto the study relatively late in the course of their disease, and so the data can, in these instances, extend for up to 20 years following the appearance of initial symptoms. It can be seen that the fitted average level of total α-syn across all of the patients tends to increase steadily with time as the disease progresses (from a value of exp(6.296) = 542.4 at t = 0 to a value of exp(7.274) = 1442 after 20 years following onset of symptoms), whereas the fitted average level of phosphorylated α-syn across the same patients remains relatively constant (with a mean value of exp(4.175) = 65.04) over many years from appearance of initial symptoms. The individual longitudinal data for each of the participants are available online (Supplementary figure 1).

### Association with gender and age

The levels of either ‘total α-syn’ or phosphorylated α-syn showed no association with gender for either the PD group or the healthy control group. The levels of these two measures of the protein also showed no correlation with sampling age in either group.

### Receiver operating curve (ROC) analysis

**Figure 4:**
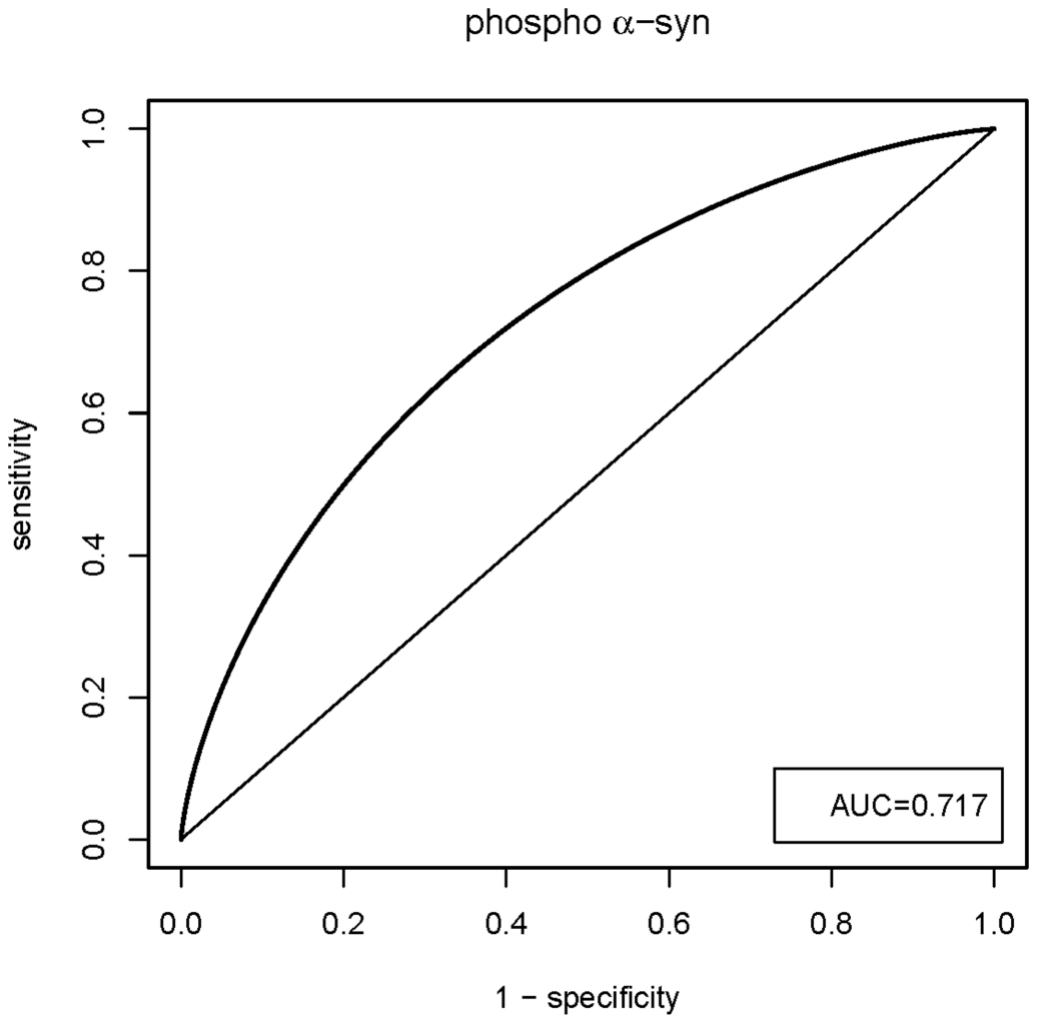
ROC curve to evaluate the utility of plasma phosphorylated α-syn levels in discriminating patients with PD from healthy controls.

Figure 4 displays an ROC curve constructed to evaluate the utility of measuring plasma phosphorylated α-syn levels as a means of discriminating between patients with PD and healthy controls. The area under the curve (AUC) gives an indication of predictive value, with AUC = 0.5 for a random association and AUC = 1 for perfect discrimination^21^. The AUC of 0.717 for phosphorylated α-synuclein is slightly better than the value of 0.68 calculated from our previously reported data with fewer participants^19^ and suggests that this protein has potential diagnostic value. On the other hand, AUC = 0.558 was obtained in the present study for ‘total α-syn’, suggesting that it has no diagnostic utility.

## Discussion

Previously, we validated novel immunoassays for determining the levels of (i) total α-syn, (ii) oligomeric α-syn, (iii) total phosphorylated α-syn and (iv) phosphorylated, oligomeric α-syn in human blood plasma and showed that all 4 of these measures are highly consistent within repeat blood samples taken at months 0, 1, 2 and 3 from the same individuals with PD, whereas they vary greatly between individuals^19^. We found that the most promising diagnostic assay was for total phosphorylated α-syn, the levels of which were numerically higher in PD samples (n = 32) than in healthy controls (n = 30), although this difference was not statistically significant (p = 0.053). Here, we have employed assays (i) and (iii) only for a more extensive follow-on longitudinal study involving 198 patients with PD (including the 32 presented in our previous report) who were assessed and sampled regularly every 4–6 months for up to 4 years from initial presentation. We chose not to employ assays (ii) and (iv) for this analysis because they were not informative in our previous study. Also, it has been reported that α-syn occurs physiologically as a helically folded tetramer^22^ which, if correct, would make the assays for oligomeric α-syn difficult to interpret because it might be impossible to distinguish between ‘normal tetramers’ and ‘pathological oligomers’.

With the extra numbers of participants recruited onto the present study (total of 198 patients with PD and 91 healthy controls) we now find that the mean level of phosphorylated α-syn is significantly higher (p = 0.012) in the PD group than in the control group, and we confirm that this is not the case for ‘total α-synuclein’ (p = 0.058). Previous studies of α-syn in blood plasma have not focussed on the phosphorylated protein and have involved only small numbers of participants and so it is not surprising that they have produced conflicting results^16^,^17^,^18^,^23^. The potential utility of phosphorylated α-syn as a diagnostic marker in plasma is reflected in the ROC analysis for this assay (AUC = 0.717) but its discriminatory ability will have to be improved for it to find genuine clinical application. There is obviously a substantial overlap between the levels of phosphorylated α-syn in PD and control plasma, suggesting that this marker alone may not be viable as a diagnostic tool. However, it is possible that genetic stratification of patients, taking into account variations within *SNCA* and other genes linked to PD, could help to explain some of the wide variation seen in α-syn plasma levels, and so lead to improvements in the utility of phosphorylated α-syn as a biomarker. Moreover, further studies are clearly required to ascertain whether the plasma levels of phosphorylated α-syn might also differ in DLB and MSA from controls and from other neurodegenerative diseases, as well as determining whether there is value in this measure as a discriminant between the different forms of α-synucleinopathies. Previous findings of ours based on post mortem cerebrospinal fluid analysis have suggested that measurement of phosphorylated α-syn might have diagnostic utility within the α-synucleinopathies^24^.

It is unclear whether phosphorylated α-syn in plasma originates mainly from a cellular component in the blood itself, or whether it originates from a peripheral tissue source elsewhere in the body, or from the brain (possibly *via* leakage or secretion/clearance across the blood-brain barrier), but this is an important area for further investigation. Phosphorylation is the most common (and probably most important) posttranslational protein modification of α-syn^25^, with Ser-129 being the main phosphorylation site. In PD, the level of soluble, cytosolic, non-phosphorylated α-syn decreases in vulnerable brain regions over the course of the disease, and the protein becomes increasingly more phosphorylated and insoluble as the disease progresses, rising from a normal level of about 5% to reach a final level of 30–100%, depending on brain region and pathological severity^26^. Phosphorylation at Ser-129 inhibits rather than promotes α-syn fibrillisation^27^ and recent data suggest that this type of α-syn phosphorylation in the brain occurs after LB formation^28^. The highly disordered periphery of the LB shows increased levels of α-syn phosphorylation compared to the interior, possibly due to the fact that the former is more accessible to phosphorylation by kinases than the latter^28^. This would suggest that α-syn phosphorylation in the brain occurs relatively late in the course of the disease, and so a brain-derived source for Ser-129 phosphorylated α-syn cannot easily explain the increased levels of this protein in blood plasma from newly affected/diagnosed patients with PD. This leads us to believe that phosphorylated α-syn in blood plasma is likely to originate from a peripheral tissue source. According to the ‘Braak staging’ hypothesis, α-syn pathology starts in the peripheral autonomic nervous system (mainly sympathetic ganglia, cardiac sympathetic afferents, and enteric nervous system) and then spreads to the CNS, with the olfactory bulb, medulla oblongata and pontine tegmentum being affected at an early (presymptomatic) stage, followed by the substantia nigra and other areas of the midbrain and basal forebrain as the disease progresses to its symptomatic stage^29^. Changes in pathological forms of α-syn in the blood plasma could reflect this temporal pattern of pathological change and, if they have a peripheral origin, provide a marker for early-stage disease. In support of this idea, post mortem histopathological studies have detected widespread phosphorylated α-syn pathology in peripheral tissues, including vagus nerve, gastrointestinal tract and endocrine organs, in PD and DLB, and to a lesser extent in incidental Lewy body disease (iLBD), which may represent a precursor to one or both of these conditions^30^.

The longitudinal analysis shows that the level of Ser-129 phosphorylated α-syn in blood plasma remains high and does not change during the course of the disease, whereas the level of ‘total α-syn’ (which would include phosphorylated and non-phosphorylated forms) tends to increase over time for up to 20 yrs after the appearance of initial symptoms. This can be best explained by a steady increase in the concentration of non phosphorylated α-syn in blood plasma as the disease progresses. Why this should be the case can only be a matter for speculation, but one possible explanation is that this reflects the increased build up of α-syn pathology and LB formation over time, and an attempt at clearance from the site of this pathology (central or peripheral) to the general blood circulation. Regardless of the explanation, our data suggest that measures of ‘total α-syn’ or possibly of ‘non phosphorylated α-syn’ could be used as a surrogate marker for disease progression in PD. This might be useful in future drug trials, especially of disease-modifying agents targeted at α-syn pathology in which the clinical goal is to slow the rate of disease progression.

## Methods

### Patient population and clinical method

Informed consent was obtained from all participants in this study. Patients with PD were recruited by DeNDRoN North West (with ethical approval from South Manchester Research Ethics Committtee) from the neurological service based at Royal Preston Hospital, along with other similar departments in the northwest of England. The diagnosis of PD was based on the UK Parkinson's Disease Society diagnostic criteria for PD^31^. Severity of disease was defined in terms of patients satisfying the criteria for stages 1 or 2 on the Hoehn and Yahr scale.

The overall target for the study was to follow a cohort of up to 200 patients meeting these criteria, over a period of 3–4 yrs from initial recruitment, reviewing them at 4–6 monthly intervals. The first 32 patients recruited onto the study were seen at monthly intervals for the first 3 months, and the results obtained from this first sampling phase have been published^19^. Blood samples were obtained at each visit, with around 3 ml of blood being collected in tubes containing EDTA. The plasma was separated within 2 hrs by centrifuging the blood at 3000 *g* for 10 mins. The plasma was immediately stored at −80°C. Appropriate care was taken to avoid contamination of the plasma samples with cells or components of the pellet obtained from the centrifugation. The samples were thawed on ice directly before analysis and repeated freeze/thaw cycles were avoided.

Control subjects were healthy individuals of similar age to those with PD with no apparent neurological or known psychiatric symptoms who were the spouses or partners of patients attending Movement Disorder or Cerebral Function Unit clinics at Salford Royal Hospital, National Health Service Foundation Trust for investigation and diagnosis of movement disorders and/or dementia. The latter control subjects were recruited as part of an ongoing investigation into the genetics and molecular biology of neurodegenerative disease approved by the Oldham Local Research Ethics Committee. Blood plasma was prepared and stored as described above by the same research staff, using the same equipment and protocol. Single blood samples only were taken from these control participants.

### Preparation of recombinant α-syn and phosphorylated α-syn protein standards

Recombinant α-syn (without any purification tag) was expressed and purified from *E. Coli* as described previously^19^. Phosphorylated α-syn was prepared from recombinant α-syn by incubation with casein kinase II^32^.

### Immunoassay methods

The sandwich immunoassay methods for the measurement of ‘total α-syn’ and Ser-129 phosphorylated α-syn in the blood plasma samples have been reported previously^19^. The assay for ‘total α-syn’ employs mouse anti-α-syn monoclonal antibody 211 (Santa Cruz Biotechnology) for capture and rabbit anti-α-syn FL-140 (Santa Cruz Biotechnology) for detection, and should detect non-phosphorylated, phosphorylated and oligomeric forms of the protein. The assay for phosphorylated α-syn utilises goat polyclonal anti-α-synuclein N-19 (Santa Cruz Biotechnology) for capture and rabbit monoclonal antibody pS129 (Epitomics) for detection. This latter immunoassay will only detect α-syn that is phosphorylated at Ser-129, and would, theoretically, include any oligomeric forms of the phosphorylated protein. Non-aggregated recombinant α-syn was used to create the standard curves required for the ‘total α-syn’ immunoassay, and non-aggregated Ser-129 phosphorylated α-syn was used to create standard curves for the phosphorylated α-syn immunoassay. The assays of blood plasma were all carried out in triplicate.

### Analysis of immunoassay data

A set of standards for one of the two different immunoassays (i.e. for ‘total α-syn’ or phosphorylated α-syn) was included on each microtitre plate, as appropriate for the type of protein being measured on that plate. Standard curves were fitted using nonlinear least squares (see Figure 1 for representative examples of standard curves for each of the two different immunoassays). The samples of blood plasma from patients with PD and controls were diluted 1 in 50 for the ‘total α-syn’ immunoassay, and 1 in 20 for the phosphorylated α-syn immunoassay. The standard curves for each individual plate were used to transform the absorbance values for that particular plate into protein concentrations, and in this way, any variation between plates was accounted for. We have already confirmed that the assay for phosphorylated α-syn does not detect the non-phosphorylated protein, and that the assay for ‘total α-syn’ will detect both phosphorylated and non phosphorylated forms of the protein^19^.

### Data analysis and statistical methods

A standard 2-sample *t* test was used to determine whether there was any significant difference between the mean levels of each of the two different forms of α-syn when comparing the plasma samples from the patients with PD with those from the healthy controls. To better satisfy the assumptions underlying this test, the empirical distributions were constructed on the logarithmic scale to obtain a more symmetrical distribution than was obtained on the original scale.

To investigate whether the protein levels changed over time, a linear mixed model was fitted to the longitudinal data from each assay, allowing for a time-trend with slope β, a random effect for patients and serial correlation amongst repeated measurements on the same patient^33^,^34^. Model parameters were estimated by maximum likelihood, and the significance of the time-trend assessed using a Wald test, i.e. under the null hypothesis of no time-trend (β = 0) the estimated value of β divided by its standard error follows a standard Normal distribution.

## Author Contributions

P.G.F. carried out the immunoassays, the cross-sectional data analysis, and wrote an initial draft of the paper. P.D. did the longitudinal statistical analysis. J.D.M. and D.M.A.M. co-ordinated the clinical aspects of the study, including blood sample collection. A.P. collected samples and data from the patients. M.H. and M.M. supplied important materials for the experiments. D.A. and D.M.A.M. conceived of the study, and D.A. revised the manuscript.

## Supplementary Material

Supplementary Information Supplementary Figure 1

## Figures and Tables

**Table 1:** Demographic details of the cohort of 189 patients with PD and 91 healthy control subjects

	Median age (Years)	Mean Age (Years)	Disease duration (years)	[total α-syn] (ng/ml)	[pS-α-syn] (ng/ml)
PD (n = 189)	62	61.9 ± 9.7	5.10 ± 4.11	1777.1 ± 3609.6	756.8 ± 2419.9
Controls (n = 91)	66	65.3 ± 9.0	N/A	1221.5 ± 2233.1	143.4 ± 531.8

The mean values for total α-syn and phosphorylated α-syn (pS-α-syn) in blood plasma are based on the first measurement taken from the patients with PD, whilst only a single blood sample was available from the healthy controls. Disease duration refers to number of years following initial symptoms, at the time of first blood sample.
