# Supplementary material for: A longitudinal study on α-synuclein in blood plasma as a biomarker for Parkinson's disease
Source: Sci Rep. 2013 Aug 29;3:2540. doi: 10.1038/srep02540 (PMC3756331; doi:10.1038/srep02540)
Supplement: Supplementary Information — Supplementary Figure 1 [file srep02540-s1.pdf]

A longitudinal study on α-synuclein in blood plasma as a biomarker for Parkinson's disease

Penelope G. Foulds[1], Peter Diggle[2], J. Douglas Mitchell[3], Angela Parker[3], Masato Hasegawa[4], Masami Masuda-Suzukake[4], David M.A. Mann[5] & David Allsop[1]

[1]Division of Biomedical and Life Sciences, Faculty of Health and Medicine, University of Lancaster, Lancaster, LA1 4AY, UK.; [2]Lancaster Medical School, Faculty of Health and Medicine, University of Lancaster, Lancaster, LA1 4AY, UK.; [3]Royal Preston Hospital, Sharoe Green Lane, Preston PR2 9HT, UK.; [4]Department of Neuropathology and Cell Biology, Tokyo Metropolitan Institute of Medical Science, 2-1-6 Kamikitazawa, Setagaya-ku, Tokyo, 156-8506, Japan; [5]Centre for Clinical and Cognitive Neuroscience, Institute of Brain, Behaviour and Mental Health, University of Manchester, Salford Royal Hospital, Salford, M6 8HD.

Correspondence and requests for materials should be addressed to D.A.

(d.allsop@lancaster.ac.uk)

Supplementary Figure 1

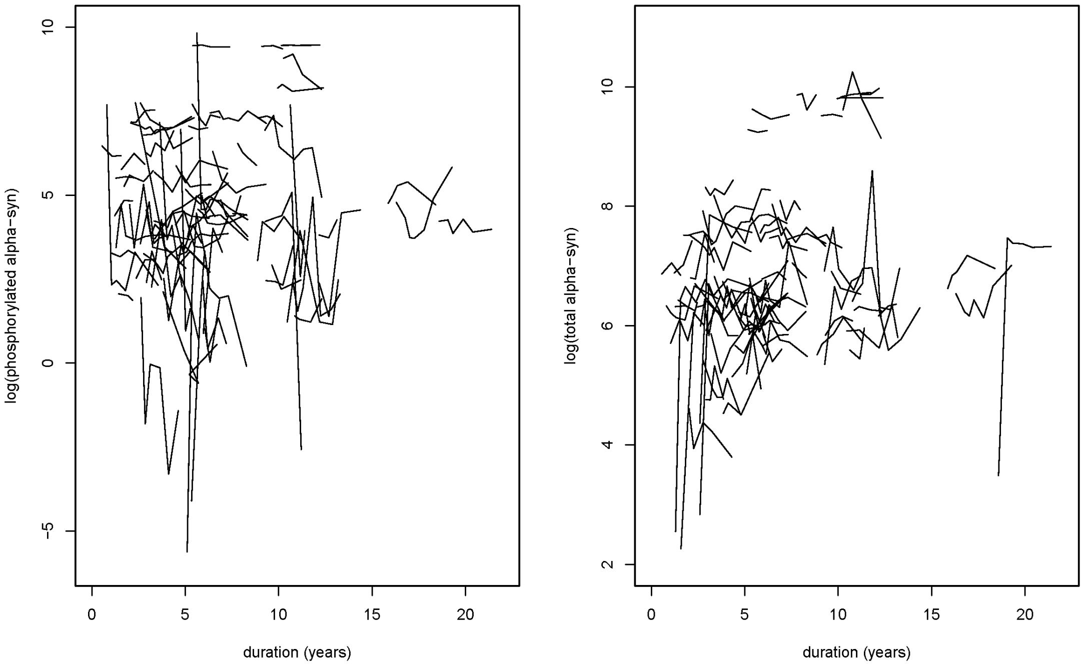

Individual longitudinal data for each of the participants with PD plotted as time since onset of initial symptoms.
